# Cognitive Intraindividual Variability and White Matter Integrity in Aging

**DOI:** 10.1155/2013/350623

**Published:** 2013-09-23

**Authors:** Nathalie Mella, Sandrine de Ribaupierre, Roy Eagleson, Anik de Ribaupierre

**Affiliations:** ^1^FPSE, University of Geneva, Boul du Pont d'Arve 40, 1211 Geneva 4, Switzerland; ^2^Clinical Neurological Sciences, Western University, 339 Windermere Road, London, ON, Canada N6A 5A5; ^3^Electrical and Computer Engineering, Western University, 1151 Richmond Street, London, ON, Canada N6A 3K7; ^4^CIGEV, University of Geneva, Boul du Pont d'Arve 40, 1211 Geneva 4, Switzerland

## Abstract

The intraindividual variability (IIV) of cognitive performance has been shown to increase with aging. While brain research has generally focused on mean performance, little is known about neural correlates of cognitive IIV. Nevertheless, some studies suggest that IIV relates more strongly than mean level of performance to the quality of white matter (WM). Our study aims to explore the relation between WM integrity and cognitive IIV by combining functional (fMRI) and structural (diffusion tensor imaging, DTI) imaging. Twelve young adults (aged 18–30 years) and thirteen older adults (61–82 years) underwent a battery of neuropsychological tasks, along with fMRI and DTI imaging. Their behavioral data were analyzed and correlated with the imaging data at WM regions of interest defined on the basis of (1) the fMRI-activated areas and (2) the Johns Hopkins University (JHU) WM tractography atlas. For both methods, fractional anisotropy, along with the mean, radial, and axial diffusivity parameters, was computed. In accord with previous studies, our results showed that the DTI parameters were more related to IIV than to mean performance. Results also indicated that age differences in the DTI parameters were more pronounced in the regions activated primarily by young adults during a choice reaction-time task than in those also activated in older adults.

## 1. Introduction

Cognitive aging is typically described in terms of a general decline of cognitive performance, in particular a general slowing of processing speed based on the mean performance of individuals [1]. More recently, it has been observed that there is an increase of intraindividual variability (IIV) with age in older adulthood [2–6]. Neuroimaging research on aging has also traditionally focused on mean group trends such as mean cognitive decline with age [7]; at this time, studies concerned with variability are still rare. As a result, little is known about the neural underpinnings of IIV. The present study aimed at investigating age differences in the relationship between cognitive IIV and the quality of the white matter, analyzing diffusion tensor imaging (DTI) and combining it with functional magnetic resonance imaging (fMRI).

Behavioral individual variability has been described in various ways, which can be summarized in four main types: (a) short-term or trial-to-trial, within-task variability, or *inconsistency*, which denotes transient and rapid fluctuations that occur over short-term time scales (e.g., within a 10-minute task); (b) intraindividual variability across tasks, or *dispersion*; (c) relatively permanent alterations that evolve slowly over relatively long-term time scales (e.g., months and years), through training, or development, that is, *intraindividual change*; (d) interindividual or between-individual variability, also termed *diversity* [3, 8, 9].

In this paper, we will focus on inconsistency, that is, on within-task short-term variability (and we will refer to this as IIV). Several studies have observed that inconsistency increases with age during adulthood and support the idea that it is a stable and meaningful indicator of individual differences [2–4, 10, 11]. Most studies have used reaction times (RT) paradigms and have observed that individual standard deviations become larger with age, even after controlling for base rate in response time. The picture is less clear with regard to accuracy-based scores: there are very few studies, on the one hand, and a number of authors did not observe an increase in IIV with age when using accuracy scores, on the other hand [12, 13]. Some studies, including ours, have also shown a U-shaped curve across the lifespan with regard to IIV, while children and older adults exhibit a larger inconsistency [14, 15]. Moreover, although “inconsistency” is usually associated with a potential dysfunction, it can also be argued that it is evidence of an adaptive dynamic process [16]. 

Despite the growth of recent studies, little is known about inconsistency in aging, and still less about its neural underpinnings. As Hultsch et al. [5] mentioned, “relative to the corpus of work based on mean level of performance, the amount of information (brought by research on IIV in cognition and aging) is miniscule” (page 547). More particularly, it is still unclear whether or not mean performance and IIV share the same neural bases [7]. Although still rare, a growing number of studies have linked IIV to brain characteristics, using either functional [17] or structural brain imaging [18–21]. Bunce and colleagues [18] showed that white matter hyperintensities in the frontal cortex were associated with larger IIV in a response time task, but not with performance in more complex cognitive tasks. Walhovd and Fjell [21] reported that IIV in response times (RTs) is correlated to white matter (WM) volume, while mean RTs are more linked to cortical grey matter (GM) volume. Recent DTI studies [19, 20] investigated the link between cognitive IIV and the quality of white matter. Comparing the influence of GM and WM on IIV in young and older adults, Moy and collaborators [20] observed that only WM had an effect on the age-related increase of behavioral inconsistency. Among the DTI parameters, only fractional anisotropy (FA) was related to cognitive variability once age as a factor had been controlled. In contrast, controlling for mean performance, Fjell and collaborators [19] observed significant relations between all DTI parameters and inconsistency in a large sample of healthy individuals. They also suggested that cognitive variability, which correlated more strongly with these parameters than the median performance, may prove to be a better correlate of changes in WM structure; the strength of these associations increased in older adults. One hypothesis for this relationship, proposed in the field, is that the loss of myelin occurring with aging causes disruptions in the efficiency of the conduction of the action potential along the axon, and WM alterations may thus be a possible mechanism to account for intraindividual variability in reaction times [22].

The objective of the present study was to further explore the relationships between IIV and the quality of the white matter, by combining DTI and fMRI in young and older adults. Regions of interest (ROI) were defined in two ways: by inspection of age differences in regions activated in a simple reaction time paradigm administered in the scanner, and by the usual means of a dedicated protocol (John Hopkins University (JHU) white-matter tractography atlas-JHU, http://cmrm.med.jhmi.edu/) [23, 24]. Several WM parameters were analyzed: fractional anisotropy (FA), mean diffusivity (MD), radial diffusivity (RD), and axial diffusivity (AD) [25]. FA refers to the coherence of the orientation of water diffusion, a higher value corresponding to a more consistent diffusion orientation, while MD is the average amount of water diffusion, a higher value denoting increased rate of diffusion. AD and RD correspond to the primary and perpendicular directions, respectively. DTI aging research traditionally reports a decrease in FA with age and an increase in other diffusivity parameters [25–30]. Some studies also have reported higher sensitivity of RD than of AD to aging [31, 32], while RD has been associated with myelin breakdown [33, 34]. In the present study, these DTI parameters were analyzed to examine their relation with inconsistency in the reaction time task administered in the scanner and also with the results obtained from a large battery of cognitive tests varying in complexity, administered in the laboratory. In accord with the literature, our hypothesis was that older adults would present a larger inconsistency than younger adults, and that DTI parameters would correlate more strongly with IIV than with mean performance.

## 2. Method

### 2.1. Participants

 Twelve young adults (aged 18 to 30 years, 10 women and 2 men) and thirteen older adults (aged 61 to 82 years, 10 women and 3 men) participated in the experiment (see Table 1 for participants' characteristics). Older adults were recruited among participants of the ongoing longitudinal Geneva Aging Study, initially recruited either from the University of the Third Age of Geneva or through newspaper and association advertisements for pensioners; most of them participated to the third wave of the study; that is, they were seen four and two years previously with the same battery of cognitive tasks; a few other ones participated for the second time, or already for the fourth time. No exclusion criteria were applied for the behavioral study, except the requirement of a good knowledge of French and of an education longer than 5-6 years. Note that the behavioral study takes place in a large university building, which requires from the participants to be independent in finding the location. A health questionnaire was also administered and showed that most older adults can be considered to be healthy. No other criteria were used for the participation to the brain imaging study, except the usual ones (no metallic implant, no claustrophobia, etc.). Young adults were comprised of a set of undergraduate psychology students who participated in the experiment as part of their course credit. All participants were fluent in French and had normal or corrected-to-normal vision. The significant differences between the two age groups (Table 1) are standard in aging studies: performance in a fluid intelligence (Progressive Matrices 38) task higher in the young adults and vocabulary or crystallized intelligence (Mill Hill) slightly higher in the older adults. Also, the number of years of education was lower in older adults than in young adults who were all university students.

The study was approved by the ethics committee of the Faculty of Psychology and Educational Sciences of the University of Geneva. All participants gave written informed consent, and older adults received a small amount of money as a compensation for their transportation fees.

### 2.2. Material and Procedure

#### 2.2.1. Behavioral Tasks

The behavioral tasks were administered in our laboratory during two sessions lasting about 1.5 hours, one week apart, for the older adults, and one session of roughly 2 hours for young adults. The older adults are part of a longitudinal study, using a rather extensive experimental protocol. The tasks presented here are a subset of this larger study. All experimental tasks were individually administered on a Dell computer, using E-Prime [35].

*The Cross-Square (CrossSquare) Task*. This choice reaction time task, adapted from Hultsch et al. [4], was administered both in the behavioral and in the fMRI sessions, in slightly different versions. In this task, participants were presented two groups of three crosses, one on the left and the other on the right part of the screen. They had to decide, as rapidly as possible, on which side a cross changed into a square. The task administered in the laboratory consisted of 120 items distributed into five blocks of 24 trials. The rate of administration depended on the participant's response rate: as soon as the response was given, the next trial appeared. The task administered in the scanner consisted of 8 blocks of 12 trials, and presentation time was fixed (1500 ms). Due to practical reasons, older adults were scanned after the behavioral session, while younger adults were scanned before the behavioral session.

*Letters Comparison Task (LetterComp).* This task of processing speed was adapted from Salthouse [36]. Participants were required to decide whether two series of letters (consisting of 6 or 9 consonants) were identical or not and had to press a *Yes *or a *No* key on the response keypad.

*Stroop Color Word Task (Stroop)*. In this widely used task [37], meant to assess resistance to interference or inhibition, participants were required to name the color in which words were displayed. Trials consisted of color words in French (“vert” (green), “jaune” (yellow), “rouge” (red), and “bleu” (blue)), and signs (””””; ++++; □□□□; ****) presented in green, yellow, red, or blue colors. This resulted in three different conditions: neutral (signs displayed in any of the four colors), congruent (color name displayed in the same color; e.g., “red” displayed in red), and incongruent (the name and the color were different; e.g., “red” displayed in blue). The task consisted of nine blocks of 24 trials, resulting in a total of 216 test trials, the condition being counterbalanced within each block. Responses were recorded into a microphone, and the experimenter manually recorded accuracy.

In order to control for processing speed, an index of resistance to interference was computed as follows: ((mean RT in the incongruent condition − mean RT in the neutral condition)/mean RT in the neutral condition). The lower the index (relative ratio), the more resistant to interference.

*Working Memory Tasks. *Two tasks of working memory were used: the Reading Span task, a verbal task [38], and the Matrices task [39] presenting both verbal and visuospatial components. In both tasks, the difficulty level was adjusted to the participant's span, which was assessed in a first phase using an ascending span procedure in which three trials of the same difficulty were presented. The participant's span was equivalent to the highest level at which two trials out of three were executed correctly. 

In the *Reading Span task* (RSpan), participants were instructed to read a series of sentences (from two to six, depending on the participants' span) on the computer screen and to decide whether each sentence was semantically correct or not (press a *Yes* or a *No* key on the keypad), while retaining the last word. Recall of the last words in their order of presentation was asked at the end of each series. The test consisted of twenty trials—ten at the participant's span level (e.g., ten series of 4 sentences) and ten at the span level +1 (ten series of 5 sentences). Response times for the semantic judgment were recorded, and the order and accuracy of oral responses were recorded manually by the experimenter.

The *Matrices task (Matrices)* consisted of a 5 × 5 grid containing either a number of blackened cells (visuo-spatial condition) or of words written within the cells (verbal-spatial condition). The number of black cells or of words ranged from two to eight, depending on the participants' span. In the visuo-spatial condition (simple positions), participants had to recall the position of the black cells and to replace them on a blank grid using a touch screen. In the verbal-spatial condition (words in the cells), participants had to recall both the words (oral response manually recorded by the experimenter) and their positions (touchscreen, response recorded by the computer). The visuo-spatial condition consisted of ten trials at span level and ten trials at span level +1; the verbal-spatial condition comprised ten trials at span level +1 and ten trials at span level +2. 

The number of correctly recalled words in the RSpan, regardless of their order, and the number of correctly recalled positions and words in the Matrices task were used as scores in the present study.

For reaction times (RTs) tasks, participants gave their response on a response pad with the forefinger of either hand (right/left or same/different), except for the Stroop Color Word task, for which a voice key was used. Accuracy in the working memory tasks was recorded by the experimenter. Several practice trials were given prior to each task (from 6 to 10 depending on the task complexity). In addition to these tasks, measures of fluid (PM 38, Raven) [40] and crystallized (Mill Hill Vocabulary Test, Deltour) [41] intelligence were assessed.

#### 2.2.2. fMRI and DTI Procedure

Participants were scanned in a Siemens Trio 3T magnet. They underwent one fMRI task sequence, as well as two fMRI sequences in a resting condition (not used in the present study), and two DTI sequences. The BOLD fMRI task-rest sequence was administered first, using a reaction time paradigm, where the participant had to indicate on which side a cross was changing into a square, as fast as possible (see CrossSquare above). The task consisted of eight experimental blocks, interspersed with eight resting blocks (resp., 52 seconds–20 seconds). The BOLD activity was obtained using an echo planar imaging acquisition (TE = 36 ms, TR = 2100 ms, flip angle = 80°, and FOV = 205 mm). Then a structural T1-weighted MRI was acquired (time echo (TE) = 2.27 ms, time repetition (TR) = 1900 ms, field of view (FOV) = 256 mm, and voxel size 1.0 × 1.0 × 1.0 mm). Finally, two sequences of 30 directions DTI were acquired (TR = 8400 ms, TE = 88 ms, *b* value = 1000 s/mm^2^, and voxel size 2.0 × 2.0 × 2.0 mm). The two DTI acquisitions, which presented a high correlation with each other (higher than .95), were concatenated to increase the signal-to-noise ratio during the postprocessing.

### 2.3. Analyses

#### 2.3.1. Behavioral Data Preparation and Analyses

In the RT tasks, only the correct responses were retained. Outliers were removed as follows: extremely fast responses (RTs below 150 ms for CrossSquare; 200 ms for Stroop; and 500 ms for LetterComp) and extremely slow responses (RTs above 1500 ms for CrossSquare; 2000 ms for Stroop; and 5000 ms for LetterComp). As mentioned above, working memory performance was scored in terms of the number of correctly recalled items (words and/or positions) by trial.

Age group effects as well as systematic time-related effects (practice or fatigue effects) were removed from the working memory and RT data by regressing age group, item rank, block rank, and their interactions on each trial, in each task. Data were then standardized and transformed into T-scores. Mean performance and within-person variability (inconsistency) were considered for the analyses. Within-person variability was assessed by computing intraindividual standard deviations (iSDs) on the T-scores. As concerns the Stroop task, residuals were computed for the raw RTs and transformed in T-scores. Then, resistance to interference was computed (relative ratio) on these T-scores, for each block; iSD was computed on the basis of these nine block indices.

In order to diminish the number of variables to correlate with brain data, and based on the between-task correlations observed, scores were further regrouped. Two combined working memory scores were used: (a) verbal working memory, assessing verbal aspects of working memory and regrouping scores obtained in the two conditions of RSpan and in the verbal part of Matrices (i.e., number of words correctly recalled at span level +1 and span level +2 in the verbal-spatial condition); (b) spatial working memory, regrouping the four conditions of the spatial aspect of Matrices (i.e., number of correctly recalled positions at span level and span level +1 in the visuo-spatial condition and at span level +1 and span level +2 in the verbal-spatial condition). This leaves altogether five behavioral scores: (1) simple reaction time (CrossSquare); (2) complex processing speed (LetterComp); (3) verbal working memory (vWM); (4) spatial working memory (sWM); and (5) resistance to interference (int). 

Univariate analyses of variance (ANOVAs) were conducted on iSDs for each of these five dimensions. As data were residualized for age group effects, no effects of age were expected on mean performance, except possibly for the Stroop, since for this task residuals were computed on the raw RTs, and not on the index of interference.

#### 2.3.2. MRI Analyses

 Image analysis was done using FSL (http://www.fmrib.ox.ac.uk/fsl) [42]. The first three volumes were discarded to allow for signal equilibration effects. Images were realigned (motion correction), slice-timed corrected, and smoothed using an 8 mm Gaussian kernel. A high pass filter was applied to remove the low-frequency noise (HP 100 s). Masks of white matter (WM) and cerebrospinal fluid (CSF) were created for each individual; their time series were extracted and used for WM and CSF regression. For each subject, the functional images were registered to their anatomical images via a rigid transformation; then using the FSL/FNIRT registration algorithm, the anatomical images were registered to the MNI_152_T1 atlas provided with the FSL software. 

The two sessions of DTI were concatenated for each subject. Each DTI volume was registered to the T1-weighted image (patient space) using FLIRT. An eddy-current correction was done. The nonbrain tissue was removed from the image (skull-striped), and least-square fits were performed to estimate the FA and eigenvector maps. The individual FA volumes were skeletonized and transformed into a common space (the fMRI58_FA template supplied by FSL) using TBSS and FNIRT. The skeletons were binarized to reduce the partial volumes between tissues borders (threshold for FA > 0.2). At every step, the warped/coregistered volumes were inspected visually for accuracy. 

Two methods were used to define ROIs for structural imaging analyses as follows.

**Figure 1:**
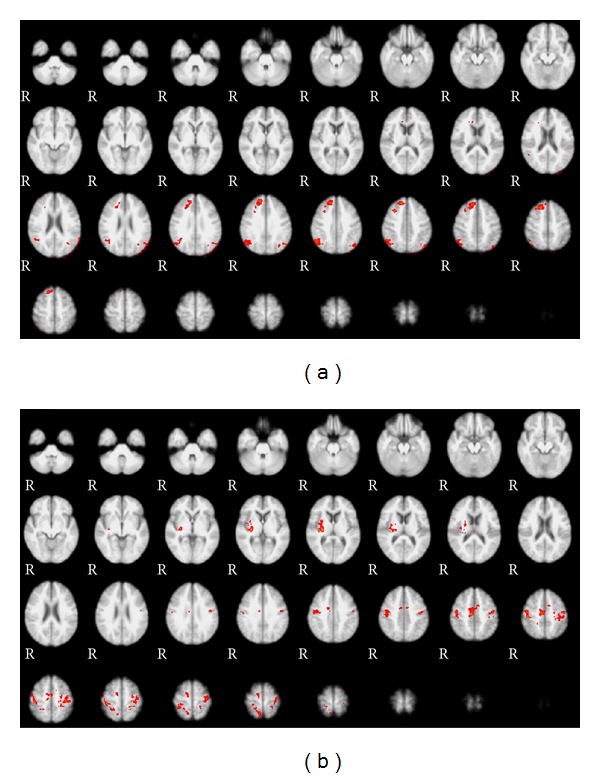
Radiological convention—right of the subject on the left of the figure. (a) Young > old: map of regions significantly more activated (in red) by the young population than by the older population in the RT task (CrossSquare). The right prefrontal (superior frontal gyrus) region is activated in the younger adults, as well as the angular gyrus (right more than left). (b) Old > young: map of regions significantly more activated (in red) by the old population than by the younger population in the RT task. There is significantly more right insular activation in the older population, as well as some larger bifrontal, supplementary motor areas and parietal networks (perirolandic).

**Figure 2:**
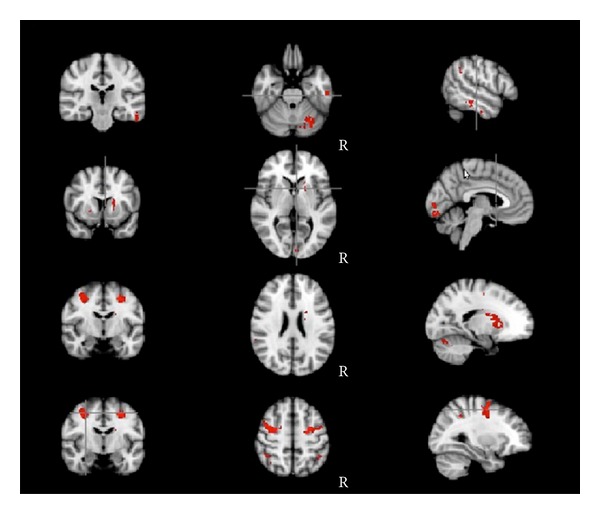
Conjunction of young and old adult activation: regions activated by both groups are highlighted in red. They comprise the right cerebellum, the right inferior temporal gyrus, the right striatum, the right occipital cortex, bifrontal rolandic cortex (hand area), and bilateral prefrontal cortex. Neurological convention—right of the subject on the axial image on the right of the figure.

(1) The first method used activated fMRI clusters as regions of interest (ROI). After a classical fMRI preprocessing, activation regions were computed as a group analysis (old versus young) contrasting the experimental and the rest blocks when the RT task was administered (cluster analysis, *Z* = 2.3, *P* = 0.05). We selected six different maps of activation on the basis of age group differences: significantly activated in young adults, significantly activated in old adults, significantly greater in young than in old (Figure 1(a)), significantly greater in old than in young (Figure 1(b)), the union, and the conjunction (Figure 2) of both groups. 

**Figure 3:**
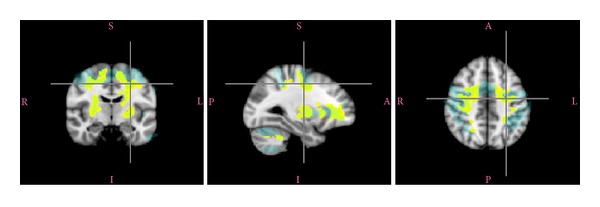
Creation of the white matter mask from the fMRI-activated areas: the yellow regions are regions activated during the contrast analysis of the young population (young only). The activated areas are then enlarged to the adjacent brain by a radius of 5 mm (blue regions). Then a mask is applied to keep the white matter only (not represented on the figure).

Then, those activation regions were enlarged (diameter 5 mm) to include the white matter surrounding them and masked, with a grey matter mask, to exclude the cortex (Figure 3). In order to map the DTI volumes to the fMRI ROI, the maps that had been created in the FA58 space were transformed to the MNI space. Finally, MD, FA, as well as AD, and RD were computed for each participant. 

(2) In order to be able to compare our data to the literature, we decided to use an atlas that had previously been used in aging studies and to apply it to our data [43–46]. Therefore, the second method used to compare the 2 groups was to coregister each brain to the JHU atlas [23, 24], and use the JHU labels to extract the FA values from each region. A mean value was also computed across the 20 regions defined in the atlas (listed in Table 2), for each of the four DTI parameters.

**Figure 4:**
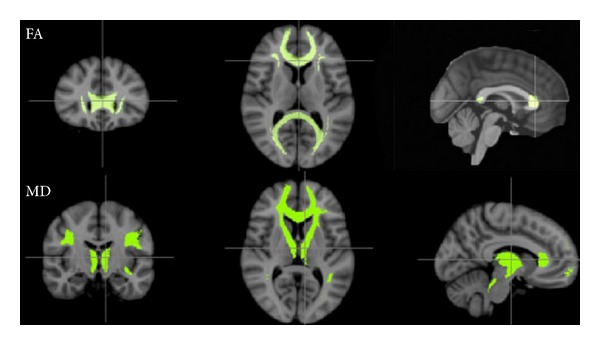
Using the JHU atlas, diffusivity measures were computed for both groups (young and old adults). The upper row represents the regions where the FA values were higher in the younger adults (forceps major and minor, bilateral inferior fronto-occipital fasciculus, and bilateral inferior longitudinal fasciculus). Mean values across areas: .478 (SD = .015) versus .448 (.012). The lower row represents the regions where the MD values were higher in the older population (bilateral anterior thalamic radiation, forceps minor, left IFO, and bilateral superior longitudinal fasciculus). Mean values across regions: .00075 (.00002) versus .00084 (.00006).

For both methods of ROIs definition, one-way analyses of variance (ANOVAs) were conducted on each of the four DTI parameters (FA, MD, AD, and RD) with age group as a between factor to assess age-related differences in the white matter quality (Figure 4). Bonferroni correction for multiple comparisons was applied. Furthermore, the relationship between all DTI parameters and the five behavioral scores described above were investigated. Correlations were computed with both iM and iSD. 

## 3. Results

### 3.1. Behavioral Results

As expected, ANOVAs conducted on mean performance for simple RTs, complex RTs, verbal working memory, and spatial working memory yielded no significant results. Note, however, that raw results (i.e., before regression and residualization) did present significant age differences in all tasks, as usual in cognitive aging studies: young adults were faster and less variable in RT tasks and had better performances in the spatial working memory task; they were, however, not significantly better in the RSpan task. Regarding resistance to interference, results showed a significant main effect of age group, (*F*_(1,23)_ = 7.233; *P* = .013; *η* ^2^ = .24), older adults showing less resistance to interference than younger adults. 

ANOVAs conducted on iSDs revealed that younger adults were less variable than older adults regarding RTs in simple tasks (*F*_(1,23)_ = 24.346; *P* < .001; *η* ^2^ = .51), RTs in complex processing speed tasks (*F*_(1,23)_ = 7.89; *P* = .01; *η* ^2^ = .25), and in spatial working memory (*F*_(1,23)_ = 6.858; *P* = .015; *η* ^2^ = .23). Within-person variability was not significantly different between age groups in verbal working memory (*F*_(1,23)_ = 1.05; *P* = .749; *η* ^2^ = .005), nor in resistance to interference (*F*_(1,23)_ = 0.135; *P* = .717; *η* ^2^ = .006). 

### 3.2. Structural Imaging Results

#### 3.2.1. ROI Based on fMRI Activation

We analyzed the FA values of the young and old participants, taking the regions of activation identified by the fMRI analysis of the contrast between the experimental and the rest blocks. The activation regions presented a slightly different pattern of activation for each group (young and old adults). The older adults presented greater activation in the right insula and in larger bifrontal/biparietal regions than the younger adults. 

**Figure 5:**
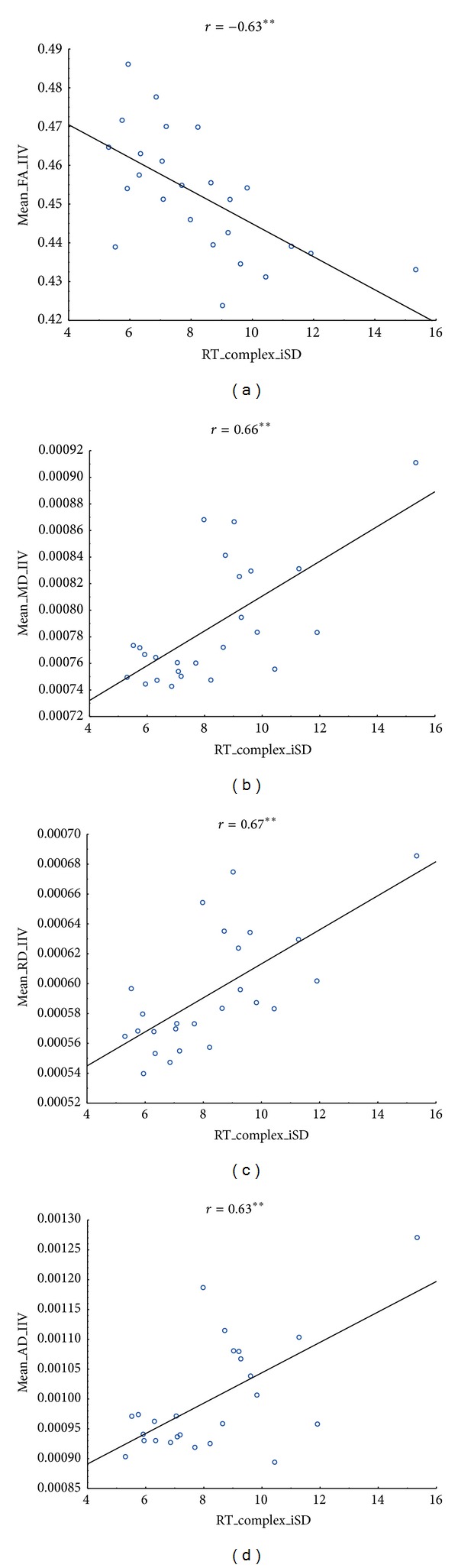
Scatterplots of iSD in the complex processing speed task and DTI characteristics (FA, MD, RD, and AD). For illustration, only ROIs showing significant correlations with IIV have been considered. IIV in processing speed was negatively related to fractional anisotropy and positively related to diffusivity (MD, AD, and RD).

As mentioned above, we analyzed six different networks: the regions activated (1) by the young adults (analyzed as a group, regardless of the older adults), (2) by the older adults (analyzed as a group, regardless of the young adults), (3) more strongly in the young than in the older adults (young > old—Figure 1(a)), that is, regions that were activated significantly more in the young than in the older adults group, (4) more strongly in the older adults than in the young adults (old > young—Figure 1(b)), (5) both by young and older adults (conjunction of regions activated both by young and older adults—Figure 2), and (6) by young or by older adults altogether (union of the two separate networks (young + old), therefore including every region activated either by the young or by the older adults). 

The regions activated by the two groups were different, with young adults activating more right prefrontal cortex, and older adults activating the right insular region as well as broader bilateral posterior frontal and parietal areas. The regions activated by all participants were the right fronto-orbital cortex, the caudate nuclei bilaterally, the superior frontal gyrus bilaterally, the supramarginal gyrus bilaterally, the inferior temporal gyrus bilaterally, the lingual gyrus bilaterally, and the left cerebellum. The regions significantly activated by older adults only were the right insula, the anterior part of the cingulum bilaterally, the superior parietal lobule bilaterally, and the right superior temporal gyrus. The regions significantly activated by the younger adults only were the right paracingulate gyrus (more anteriorly than the cingulate activation in the older adults), the left middle temporal gyrus, the right angular gyrus, and the right middle frontal gyrus anteriorly. 

Finally, each activated area was enlarged to capture the WM adjacent to it; then the cortex was excluded using a mask since diffusivity values are only interesting in white matter. 

#### 3.2.2. Age Effects on White Matter Quality

Results showed significant effects of age in regions exclusively activated in young adults in FA (*F*_(1,23)_ = 14.77; *P* = .001; *η* ^2^ = .40), MD (*F*_(1,23)_ = 13.403; *P* = .001; *η* ^2^ = .38) and RD (*F*_(1,23)_ = 19.405; *P* < .001; *η* ^2^ = .47). In addition, age effects were observed in regions activated more by young adults than by older adults in both MD (*F*_(1,23)_ = 12.558; *P* = .002; *η* ^2^ = .36) and RD (*F*_(1,23)_ = 11.647; *P* = .002; *η* ^2^ = .35). RD in regions only activated by both groups (union) also showed an age effect (*F*_(1,23)_ = 9.076; *P* = .006; *η* ^2^ = 29). 

No age effects were observed for the DTI parameters either in regions activated by older adults (exclusively or more powerfully than in young adults) or in the conjunction of the regions activated by both young and older adults. 

#### 3.2.3. Correlations with Behavioral Data

We only examined correlations with the CrossSquare (RT) task, that is, the task for which regions of functional activation were analyzed, whether administered in the scanner or in the laboratory. With regard to CrossSquare administered in the laboratory, there was a significant negative correlation between iSD and FA (−0.47), as well as a positive correlation between iSD and RD (0.46); both DTI values were obtained in the regions activated by the young adults only. However, those results were not significant in the same task repeated in the magnet.

### 3.3. ROIs Based on JHU-Atlas (20 Regions)

#### 3.3.1. Age Effects on the White Matter Quality

Table 2 displays age effects observed in the white matter quality. Results showed age differences in FA in forceps major, forceps minor, bilateral inferior fronto-occipital (IFO) Fasciculus, and right inferior longitudinal fasciculus (ILF), the young adults showing significantly more FA in these regions than the older adults. The mean FA value, computed across the 20 regions, was also higher in young adults. Mean diffusivity was significantly larger in older adults in bilateral anterior thalamic radiation (ATR), left IFO, and bilateral superior longitudinal fasciculus (SLF), as well as for the overall mean value. Radial diffusivity showed similar age effects, in the same regions as MD, as well as in the left cingulum/cingulate gyri, the forceps minor, and the right IFO. In contrast, axial diffusivity showed age effects only in left and right ATR and left SLF, with older adults presenting higher values. 

#### 3.3.2. Correlations with Cognitive Measures

Correlation analyses were carried out between the four DTI parameters and mean performance, on the one hand, and between the four DTI parameters and iSDs, on the other hand, for the tasks administered in the laboratory.  Table 3 reports the significant correlations observed for FA. Correlations with the other DTI parameters are in Tables 4, 5, and 6; see also Figure 5. Results showed significant negative correlations between within-person variability (iSD) in the complex RT task and FA in a number of ROIs, in particular those presenting age differences (between *r* = −.40 and *r* = −.72, all *P* < .05). A similar relation was observed for iSD in the simple task and FA in the forceps minor (*r* = −.50, *P* < .01) and between iSD in spatial working memory and FA in bilateral IFO (*r* = −.45, *P* < .05 and *r* = −.46, *P* < .05). A few correlations were also significant between mean performance and FA values. iM in the complex processing speed task was positively related to FA in bilateral SLF (temporal part) (*r* = −.43, *P* < .05 and *r* = −.46, *P* < .05); and mean level of resistance to interference significantly correlated with FA in the forceps minor and in the left cingulum (*r* = −.41, *P* < .05).

With regard to the mean and radial diffusivity (MD and RD), our results also showed strong significant correlations between the degree of diffusivity and iSD in most tasks. Cognitive intraindividual variability was positively and significantly related to MD and RD in all tracks (except in bilateral IFO) for the complex RT task (between *r* = .42, *P* < .05 and *r* = .68, *P* < .01), and in most tracks for the simple RT task (between *r* = .41, *P* < .05 and *r* = .55, *P* < .01), with the exception of left IFO for MD, and bilateral ILF, and forceps minor for RD. Variability in spatial working memory also correlated significantly with RD in left IFO and left CS (*r* = .42, *P* < .05 and *r* = .42, *P* < .05, resp.). Fewer significant correlations between mean performance, on the one hand, and mean or radial diffusivity, on the other hand, were observed (see Tables 4 and 5). 

Results concerning axial diffusivity (AD) were slightly different (see Table 6). They displayed more correlations with iM performance, notably as concerns the verbal working memory (between *r* = −.42, *P* < .05 and *r* = .54, *P* < .01). Interestingly, all significant correlations were with tracks showing no age differences in AD. In tracks showing age differences, AD was significantly related to cognitive IIV in both simple and complex RT tasks (between *r* = .42, *P* < .05 to *r* = .58, *P* < .01), and positively related to mean performance in both simple and complex RT tasks in bilateral ATR (*r* = .41, *P* < .05). 

## 4. Discussion

This study aimed to assess age differences in the quality of white matter and to explore its relationships with cognitive performance; in particular, the “inconsistency” (i.e., within-task intraindividual variability, abbreviated as IIV). WM was studied using diffusion tensor imaging (DTI), by using two methods to define relevant regions: regions defined by age differences in functional activation in response to a choice reaction time (RT) task and regions defined a priori on the basis of the JHU atlas. Four DTI parameters were computed: fractional anisotropy (FA), mean (MD), radial (RD), and axial (AD) diffusivity. 

### 4.1. Age Differences in WM Quality

On the basis of fMRI, six sets of regions were defined: significant activation in younger adults, significant activation in older adults, significantly greater activation in younger than in older adults, significantly greater activation in older than in younger adults, regions significantly activated in both age groups (conjunction), and regions activated either by one group or the other (union). Interestingly, age differences in FA, as well as in MD and RD, were only significant for those regions more strongly or uniquely activated in young adults; FA values were higher in young adults, while MD and RD values were higher in older adults. In contrast, there were no age differences in those regions which were also strongly activated in older adults. We will return to this point below. On the basis of the JHU atlas, 20 regions were defined and a mean value was computed, for each of the four DTI parameters. When presenting a significant age difference, FA was higher in young adults; this was the case for forceps major and forceps minor, the bilateral inferior fronto-occipital fasciculi (IFO), as well as the right inferior longitudinal fasciculus (ILF), and the overall mean. MD, RD, and AD indices, when differing between the two groups, were higher in older adults, and this concerned the bilateral anterior thalamic radiation (ATR) and the bilateral superior longitudinal fasciculi (SLF—only the left one for AD). MD and RD were higher in the left ILF; RD showed additional differences: left cingulum/cingulate gyri, right IFO. MD and RD mean values were also significantly higher. 

Our results converge with those of Madden and colleagues [28] who showed, focusing on average values rather than IIV, a decrease in FA in the forceps major and minor and an increase in RD in the forceps minor for the older population compared to the younger adults. However, our results do not show the additional increase in RD in the forceps major or the decrease in the AD for the Forceps minor described by Madden and collaborators. The fact that areas activated by the young people only, and not by the older adults, showed a significant decrease in FA in the older adults as well as an increase in MD and RD, is compatible with demyelination found in animal models [47]. Differences in FA associated with differences in RD, but without differences in AD, as seen in most of our study when comparing the two age groups, might fit under the first pattern described by Bennett et al. [25] and be related to microstructural changes instead of macrostructural ones.

We also observed a small anterior posterior gradient (with more differences occurring in the frontal areas, forceps minor, and ATR, rather than posteriorly) as seen in the literature and explained by an increased susceptibility of the anterior white matter to age-related alterations [25, 48–50]. This has also been observed by Madden and colleagues [27] who showed age differences in prefrontal areas for both FA and MD values.

### 4.2. Age Differences in Cognitive IIV

The behavioral performance was studied by means of a choice reaction time task administered in the scanner (CrossSquare task), on the one hand, and by a battery of several cognitive tasks, varying in complexity, administered in our laboratory: reaction time (CrossSquare), more complex processing speed (LetterComp), inhibition (interference score in the Stroop task), verbal working memory (a score combining RSpan and Matrices), and spatial working memory (memory for positions in the simple and double Matrices task, on the other hand). Age differences were observed in all the tasks as concerns the mean raw scores. However, the focus was placed here on residual scores, once age and trial and block order (practice effect), as well as their interactions, had been controlled [5]. Obviously, no age differences were to be expected any longer on the mean residualized scores, but only, if any, in the intraindividual standard deviation computed on these residual scores (iSDs). Such age differences in IIV were indeed observed in most tasks, showing a larger variability in older adults than in younger adults. Two scores did not present a significant age difference in IIV: verbal working memory and interference in the Stroop task. In the latter task, note, however, that older adults were found to be altogether more sensitive to interference, when computed as a relative ratio to control for differences in speed. As concerns verbal working memory, there was no age difference, whether in terms of mean performance or of IIV. This result can be interpreted in two, nonexclusive manners. First, as mentioned in the introduction, the finding of a larger IIV in older adults has been observed essentially in RT tasks, but not as systematically as concerns accuracy [12, 13]. The present results also point to this nonsystematicity, as the spatial, but not the verbal working memory, does present a higher IIV in older adults. A second reason for this difference between verbal and spatial working memory can also be found in the overall smaller age effects usually observed in verbal than in spatial or fluid intelligence tasks [51, 52]. This effect is well known as concerns the average performance, but has not yet, to our knowledge, been observed with respect to IIV. Therefore, this will merit replication with a larger sample.

### 4.3. Relation between Cognitive IIV and WM Quality

Globally, quality of WM was mostly related with IIV and not with mean performance (at least when basic rate in response times has been controlled for, as in iM and iSD). Variability in the Letter Comparison task was negatively related to FA in almost all regions and was positively related to MD and RD in regions showing age differences. IIV in the Cross Square task performed outside the scanner correlated with FA only in the forceps minor and with MD and RD in bilateral ATR and SLF, in addition to the cingulum/cingulate gyrus for RD. It is true, as one of the reviewers rightly commented, that the difference in correlations was not directly tested. Note, however, that the sheer number of significant correlations was clearly higher with IIV than with the mean, in particular as concerns the FA values. These observations are consistent with Fjell and collaborators' report of a stronger relation between WM quality and IIV than with average performance [19]. They also provide additional support to the hypothesis that reduced WM structural integrity generates increased neural noise and performance variability [53, 54]. 

The relationship between WM quality and IIV in simple RT tasks was, however, less important than in the complex processing speed task. Complex cognitive tasks typically involve increasing interplay between multiple areas, spatially distributed [55, 56]. Progressive breakdown in myelin with aging may yield neural noise and less stable communication between areas, hence accounting for an increase of IIV in reaction times [21, 22]. Therefore, the more spatially distributed the areas, or/and the more complex the tasks, the more IIV should be observed. This might explain the stronger relation observed with complex processing speed tasks than with simple RT tasks. However, complexity does not explain the difference in result in correlational pattern between the CrossSquare task performed in the scanner and the CrossSquare task performed in the laboratory, for which we do not have a ready explanation; only the latter did correlate with the DTI parameters (only with the forceps minor as concerns FA, and with more regions with regards to MD and RD). The two versions of the task were indeed not fully identical, but very similar. They differed in the number of trials (96 in the scanner versus 120 in the laboratory), and, perhaps more importantly, in the rate of presentation: it was adapted to the participant's response in the laboratory, but fixed (1500 m sec) in the scanner. Moreover, the order was also different: laboratory then scanner for the older adults, but the reverse for young adults. Note, however, with respect to this last point, that age effects were observed in both conditions. Finally, it should be reminded that, when focusing on the within-magnet results, IIV was correlated with the activated regions on which the DTI parameters were calculated (in the six networks described above). In contrast, when focusing on the laboratory results, the DTI parameters were calculated on the JHU 20 regions. These two sets of regions were not exactly the same; obviously the individual differences assessed by correlations also differ somewhat. Before attempting to understand this intriguing finding, and comparing more finely the two sets of regions (activated regions versus JHU ones), we prefer waiting for a larger sample to be completed (see below).

If IIV does increase with complexity, one might also expect a significant relation between DTI parameters and working memory tasks, which are certainly more complex than our RT tasks. This was not the case in this study. IIV in spatial working memory did correlate with FA in bilateral IFO, as well as with RD in the left IFO and left CS tracks, but IIV in verbal working memory showed no correlation with any of the DTI parameters. As already mentioned above, IIV might behave differently depending on whether the cognitive performance is scored in terms of RT or of accuracy. Also, verbal performance does not present the same aging pattern, altogether, as other cognitive abilities such as speed or spatial cognition. Walhovd and Fjell [21] reported a negative correlation of WM volume with performance abilities and processing speed, but no relation with verbal abilities. Note, also, that processing speed might have played a more important role in spatial working memory, as presentation rate was timed in the Matrices task, whereas it was individually adjusted in the RSpan task. The relation between working memory abilities and processing speed is not fully elucidated yet [57] and seems to change over the lifespan [15, 58]. The present correlational results, together with the lack of age differences in DTI parameters in a number of regions, also point to heterogeneity in the aging processes [59].

Note that, as mentioned above, other studies also report a link between mean performance and DTI parameters [28, 31, 48, 60]. For example, Madden and collaborators [28, 48] report a negative correlation between RT and FA values in the frontal lobe or anterior part of the corpus callosum in older adults. Davis and colleagues [31] observed an effect of FA in older adults showing a better performance when the FA was elevated. Kennedy and Raz [60] also observed a correlation between processing speed and FA in the anterior part of the brain (prefrontal cortex) but not posteriorly, which is compatible with our results in the Letter-Comparison test (a negative correlation between variability and FA value). Therefore, WM quality seems to be related with both IIV and mean performance but the relations might not be exactly the same. 

### 4.4. Limitations and Future Research

 Obviously, the present study is not without some limitations. First, as already mentioned, the sample is still small, and results await replication with a larger number of participants. Because the older participants are examined within a broader, longitudinal study, data collection is still ongoing and has to wait for a given time interval before a new assessment can be conducted. Therefore, increase in the sample size takes time to evolve within the population being studied. It is worth stressing that significant associations were already observed with a small sample size in the present study and that the effects' size was often large. Nevertheless, an increase in the sample size should help clarifying a few issues, or, in the case of somewhat different results, would point to the importance of interindividual differences. Second, as just mentioned, although yielding interesting results, the analysis of region-based DTI parameters based on brain activity is task dependent. The results in terms of age differences might differ if the task used in the scanner was more complex. It is, however, difficult to adopt a more extensive protocol in the scanner. Future analyses, based on a larger sample, will focus on the relationship between white matter and observed change over the three waves to which the older adults were submitted. Lastly, some studies have shown that IIV was a strong predictor of cognitive decline, including mild cognitive impairment or dementia [5, 10, 61]. Regarding our results, one may assume that the quality of the WM plays a role in the relation between IIV and pathological cognitive decline. Our results also draw attention to the role of individual differences. They are very large within a given age group, whether in average performance or in terms of inconsistency and intraindividual change in cognitive behavior (as shown by our longitudinal study on inconsistency), or whether in terms of brain activation and white matter quality. They should be granted more importance before any conclusion with respect to pathological aspects of brain and cognitive aging can be drawn.

To summarize, the present study provides further empirical support to the proposal that IIV in cognitive behavior increases with age [5] and brings complementary information to the work based on mean level performance. Despite controlling for age group, order of trials, and of blocks, IIV was higher in older adults in most tasks. Accordingly, with our hypotheses, IIV also correlated with DTI parameters, in particular in those regions showing age differences. Congruent with Fjell and collaborators' results [19], FA and MD correlated more strongly with iSD than with the mean performance. An intriguing result concerns the age differences in WM in the functionally activated regions: such age differences were only observed in the areas more strongly or exclusively activated in young adults. In contrast, the DTI parameters did not present age differences in the zones more strongly activated in the older adults. If reliable, this finding might be understood in terms of the compensation-dedifferentiation model [62, 63]. Due to a change of WM microstructure in some brain areas in older adults—reflected in a decrease in FA values or anisotropy—, those areas might be less functional. When confronted with a given task, older adults might rely upon different regions than the young adults, less sensitive to a breakdown in myelin. Altogether, our results are also compatible with the assumption that behavioral IIV, more than mean performance, is linked with a loss of WM integrity. 

## Figures and Tables

**Table 1:** Participants' demographic information.

	Young	Old	Age effects*
Age	21.58 (3.83)	69.85 (5.64)	<.001
Mill Hill	32.25 (2.83)	37.15 (5.58)	.04
PM38	51.50 (3.23)	39.15 (8.72)	<.001
Education level	17 (1.73)	9.61 (1.45)	<.001

Mean values (standard deviation). *P values.

**Table 2:** Age effects on white matter quality in the JHU-ROIs.

JHU-ROIs	Effect sizes^a^			
	FA	MD	RD	AD
Anterior thalamic radiation L	.186	.414*	.411*	.409*
Anterior thalamic radiation R	.247	.362*	.366*	.348*
Corticospinal tract L	.112	.157	.265	.000
Corticospinal tract R	.119	.142	.269	.010
Cingulum/cingulate gyrus L	.171	.285	.336*	.009
Cingulum/cingulate gyrus R	.087	.083	.153	.005
Cingulum/hippocampus L	.020	.007	.001	.019
Cingulum/hippocampus R	.005	.027	.027	.013
Forceps major	.336*	.050	.080	.018
Forceps minor	.634**	.254	.448**	.011
Inferior fronto-occipital fasciculus L	.494**	.356*	.493**	.102
Inferior fronto-occipital fasciculus R	.402*	.213	.359*	.019
Inferior longitudinal fasciculus L	.324	.155	.271	.004
Inferior longitudinal fasciculus R	.380*	.027	.157	.057
Superior longitudinal fasciculus L	.181	.502**	.529**	.420**
Superior longitudinal fasciculus R	.225	.464**	.566**	.176
Uncinate fasciculus L	.211	.132	.226	.002
Uncinate fasciculus R	.071	.083	.113	.011
Superior longitudinal fasciculus temporal part L	.077	.000	.034	.058
Superior longitudinal fasciculus temporal part R	.002	.022	.015	.006
Mean value	.324*	.311*	.425**	.127

Values in the table represent effect sizes. L: left; R: right. FA: fractional anisotropy; MD: mean diffusivity; RD: radial diffusivity; AD: axial diffusivity.

^a^Effect sizes are partial eta squared (*η* ^2^).

*P < .0025; **P < .0005.

For the mean values, *P < .005; **P < .001.

**Table 3:** Correlations between FA in the JHU-ROIs and cognitive measures.

JHU-ROIs	CrossSquare		LetterComp		vWM		sWM		int	
	iM	iSD	iM	iSD	iM	iSD	iM	iSD	iM	iSD
Left ATR	.05	−.31	−.31	**−.72** ^**∗∗**^	.20	−.01	−.07	−.23	−.15	−.15
*Right ATR *	*.01 *	*−.31 *	*−.36 *	**−.72** ^**∗∗**^	−.03	*−.14 *	*−.02 *	*−.20 *	*−.31 *	*−.06 *
Left CS	.20	−.07	.12	−.38	.28	−.01	−.15	−.38	−.09	−.04
Right CS	.15	−.06	.09	−.35	.35	−.05	.02	−.21	−.04	−.09
Left Cing	−.08	−.38	−.34	**−.59** ^**∗∗**^	.10	−.27	−.08	−.14	**−.41** ^**∗**^	.00
Right Cing	−.02	−.24	−.22	**−.47** ^**∗**^	.16	−.16	−.13	−.12	−.24	−.05
Left Cing_h	.32	.17	.06	−.17	.09	.03	−.25	.02	−.01	.07
Right Cing_h	.02	.07	−.02	−.17	.16	−.12	.14	.04	.09	.26
*Forceps major *	*.08 *	*−.29 *	*−.12 *	***−.57*** ^***∗∗***^	*.26 *	*.12 *	*−.10 *	*−.36 *	*−.10 *	*.02 *
*Forceps minor *	*−.02 *	***−.50*** ^***∗***^	*−.21 *	***−.63*** ^***∗∗***^	*−.01 *	*−.11 *	*−.08 *	*−.30 *	***−.41*** ^***∗***^	*.00 *
*Left IFO *	*.24 *	*−.35 *	*.06 *	***−.49*** ^***∗***^	*.06 *	*−.09 *	*−.16 *	***−.45*** ^***∗***^	*−.25 *	*−.04 *
*Right IFO *	*.39 *	*−.22 *	*.02 *	***−.48*** ^***∗***^	*−.09 *	*−.14 *	*−.27 *	***−.46*** ^***∗***^	*−.23 *	*.03 *
*Left ILF *	*.26 *	*−.33 *	*.06 *	***−.40*** ^***∗***^	*.16 *	*−.08 *	*.05 *	*−.22 *	*−.16 *	*−.04 *
*Right ILF *	*.29 *	*−.24 *	*−.04 *	***−.55*** ^***∗∗***^	*.11 *	*−.05 *	*.01 *	*−.29 *	*−.27 *	*−.11 *
Left SLF	.03	−.27	.05	−.38	.10	.04	−.03	−.26	−.26	−.05
Right SLF	.02	−.31	.03	**−.41** ^**∗**^	.18	.06	−.09	−.36	−.21	−.04
*Left UN *	*.26 *	*−.07 *	*.02 *	***−.43*** ^***∗***^	*.01 *	*−.37 *	*−.38 *	*−.35 *	*−.18 *	*−.01 *
Right UN	.15	.10	−.26	−.47*	.01	−.23	−.26	−.26	−.22	.16
Left SLF_t	.24	.08	**.43** ^**∗**^	.02	−.10	−.13	−.28	−.07	.16	.09
Right SLF_t	.19	.02	**.46** ^**∗**^	.28	.05	−.15	.01	−.01	−.11	.08
Mean FA	.23	−.24	−.01	−.58 **	.15	−.17	−.17	−.32	−.26	.03

ATR: anterior thalamic radiation L; CS: corticospinal tract; Cing: cingulum/cingulate gyrus; Cing_h: cingulum/hippocampus; IFO: inferior fronto-occipital fasciculus; ILF: inferior longitudinal fasciculus; SLF: superior longitudinal fasciculus; UN: uncinate fasciculus; SLF_t: superior longitudinal fasciculus temporal part; iM: individual mean; iSD: individual standard deviation; FA: fractional anisotropy. Tracks in italic are those showing an age effect. CrossSquare: simple RT; LetterComp: complex processing speed; vWM: verbal working memory; sWM: spatial working memory; int: resistance to interference.

*P < .05; **P < .01.

**Table 4:** Correlations between MD in the JHU-ROIs and cognitive measures.

JHU-ROIs	CrossSquare		LetterComp		vWM		sWM		int	
	iM	iSD	iM	iSD	iM	iSD	iM	iSD	iM	iSD
*Left ATR *	*.13 *	.55**	.41*	.62**	*−.03 *	*−.07 *	*.00 *	*.31 *	*.26 *	*−.09 *
*Right ATR *	.18	.50*	.41*	.60**	−.08	−.02	−.18	.17	.29	.00
Left CS	−.30	.15	.23	**.51**	−.15	−.15	.13	.23	.09	.02
Right CS	−.19	.08	.02	.31	−.30	.00	−.03	−.08	.05	−.05
Left CC	−.18	.26	.18	.55**	−.22	.11	.04	.26	.11	−.15
Right CC	−.19	.06	−.05	.30	−.48*	−.11	−.02	.14	−.07	.26
*Left Cing_h *	*−.33 *	*−.16 *	*.07 *	*.11 *	*−.33 *	*−.04 *	*.10 *	*−.09 *	*−.17 *	*−.19 *
Right Cing_h	−.08	.00	.25	.37	−.32	−.10	−.20	−.14	.06	−.14
Forceps major	−.01	.12	−.09	−.03	−.37	−.07	−.16	−.14	−.05	.10
*Forceps minor *	−.04	.13	.25	.42*	−.24	−.13	.07	.09	.10	−.09
*Left IFO *	−.22	.23	.07	.39	−.31	−.13	.05	.35	.10	−.05
*Right IFO *	−.33	.06	.06	.32	−.30	−.13	.06	.15	.03	.01
Left ILF	−.30	.11	.05	.32	−.44*	−.12	−.03	.16	.02	.03
Right ILF	−.34	−.19	−.02	.19	−.43*	−.12	.10	.00	−.15	−.04
*Left SLF *	*.02 *	.47*	*.28 *	.60**	*−.22 *	*−.14 *	*−.12 *	*.31 *	*.35 *	*.02 *
*Right SLF *	*−.08 *	.41*	*.15 *	.50*	*−.33 *	*−.09 *	*.03 *	*.26 *	*.34 *	*−.10 *
Left UN	−.06	.16	.14	.44*	−.40	−.05	.04	.28	−.04	−.19
Right UN	−.13	.00	.27	.49*	−.20	−.21	.07	.13	.03	.05
Left SLF_t	−.35	−.15	−.19	−.09	−.46*	.01	.09	−.03	−.16	−.07
Right SLF_t	−.39	−.08	−.43*	−.19	−.39	.03	.09	−.03	−.08	−.07
Mean MD	−.12	.28	.21	.50*	−.35	−.10	−.04	.16	.13	−.04

ATR: anterior thalamic radiation L; CS: corticospinal tract; CC: cingulum/cingulate gyrus; Cing_h: cingulum/hippocampus; IFO: inferior fronto-occipital fasciculus; ILF: inferior longitudinal fasciculus; SLF: superior longitudinal fasciculus; UN: uncinate fasciculus; SLF_t: superior longitudinal fasciculus temporal part; iM: individual mean; iSD: individual standard deviation; FA: fractional anisotropy. Tracks in italic are those showing a significant age effect.

*P < .05; **P < .01.

**Table 5:** Correlations between RD in the JHU-ROIs and cognitive measures.

JHU-ROIs	CrossSquare		LetterComp		vWM		sWM		int	
	iM	iSD	iM	iSD	iM	iSD	iM	iSD	iM	iSD
*Left ATR *	.13	.55**	.40*	.64**	−.02	−.05	.01	.32	.27	−.08
*Right ATR *	.18	.50*	.40*	.61**	−.06	.01	−.17	.18	.30	.00
*Left CS *	−.32	.19	.05	.51**	−.16	−.06	.19	.44*	.12	.01
*Right CS *	−.18	.17	−.06	.40*	−.31	.06	.01	.18	.11	−.03
Left CC	−.03	.41*	.31	.68**	−.15	.20	.06	.23	.33	−.08
Right CC	−.10	.20	.07	.47*	−.38	−.01	.05	.17	.11	.23
Left Cing_h	−.34	−.11	.07	.24	−.26	−.07	.17	−.02	−.06	−.18
Right Cing_h	−.03	.05	.23	.38	−.27	−.06	−.22	−.09	.06	−.18
Forceps major	−.01	.15	−.07	.06	−.37	−.08	−.13	−.07	−.04	.07
*Forceps minor *	−.02	.29	.28	.55**	−.18	−.07	.06	.20	.23	−.06
*Left IFO *	−.20	.34	.06	.47*	−.22	−.05	.07	.42*	.17	−.03
*Right IFO *	−.35	.16	.07	.43*	−.17	−.04	.13	.29	.11	−.02
*Left ILF *	−.30	.24	.04	.41*	−.36	−.06	−.05	.20	.10	.04
*Right ILF *	−.38	−.02	.00	.38	−.33	−.04	.08	.13	.02	.02
*Left SLF *	.01	.49*	.24	.60**	−.18	−.12	−.09	.32	.37	.02
*Right SLF *	−.05	.50*	.13	.54**	−.22	−.08	.04	.36	.40*	−.08
*Left UN *	−.16	.16	.09	.50*	−.25	.13	.19	.38	.06	−.15
Right UN	−.17	−.03	.29	.54**	−.11	−.06	.18	.23	.10	−.05
Left SLF_t	−.33	−.10	−.35	−.05	−.27	.05	.19	.02	−.19	−.12
Right SLF_t	−.29	−.03	−.49*	−.26	−.21	.09	.04	.01	.04	−.11
Mean RD	−.11	.35	.19	.59**	−.27	−.03	.00	.25	.21	−.05

ATR: anterior thalamic radiation L; CS: corticospinal tract; CC: cingulum/cingulate gyrus; Cing_h: cingulum/hippocampus; IFO: inferior fronto-occipital fasciculus; ILF: inferior longitudinal fasciculus; SLF: superior longitudinal fasciculus; UN: uncinate fasciculus; SLF_t: superior longitudinal fasciculus temporal part; iM; individual mean; iSD: individual standard deviation; FA: fractional anisotropy. Tracks in italic are those showing a significant age effect.

*P < .05; **P < .01.

**Table 6:** Correlations between AD in the JHU-ROIs and cognitive measures.

JHU-ROIs	CrossSquare		LetterComp		vWM		sWM		int	
	iM	iSD	iM	iSD	iM	iSD	iM	iSD	iM	iSD
*Left ATR *	.13	.53**	.41*	.58**	−.06	−.10	−.03	.28	.24	−.12
*Right ATR *	.17	.48*	.41*	.58**	−.11	−.06	−.20	.17	.26	.00
Left CS	−.12	.01	.38	.24	−.07	−.22	−.04	−.23	.00	.02
Right CS	−.09	−.13	.15	−.03	−.12	−.11	−.09	−.48*	−.08	−.05
Left CC	−.31	−.16	−.15	−.03	−.19	−.11	−.03	.12	−.33	−.15
Right CC	−.22	−.11	−.17	.00	−.42*	−.20	−.09	.06	−.25	.20
Left Cing_h	−.21	−.19	.04	−.13	−.32	.02	−.04	−.16	−.28	−.14
Right Cing_h	−.17	−.12	.21	.22	−.34	−.16	−.12	−.23	.05	.01
Forceps major	−.01	.08	−.11	−.14	−.35	−.06	−.21	−.23	−.06	.13
Forceps minor	−.08	−.19	.15	.10	−.29	−.21	.07	−.13	−.16	−.13
Left IFO	−.22	.02	.08	.19	−.43*	−.25	.01	.18	−.06	−.09
Right IFO	−.23	−.11	.03	.06	−.46*	−.25	−.05	−.12	−.11	.06
Left ILF	−.22	−.14	.06	.09	−.45*	−.20	.02	.03	−.12	.00
Right ILF	−.16	−.37	−.05	−.16	−.42*	−.20	.10	−.20	−.33	−.11
*Left SLF *	.03	.42*	.35	.58**	−.29	−.17	−.17	.27	.29	.01
Right SLF	−.13	.18	.16	.33	−.44*	−.10	.01	.03	.18	−.12
Left UN	.12	.12	.17	.19	**−.50**	−.34	−.23	.01	−.20	−.20
Right UN	−.03	.06	.16	.25	−.31	−.44*	−.15	−.08	−.10	.22
Left SLF_t	−.25	−.16	.08	−.10	−.54**	−.04	−.08	−.08	−.06	.02
Right SLF_t	−.25	−.10	.05	.09	−.39	−.11	.10	−.09	−.22	.06
Mean AD	−.11	.12	.21	.28	−.45*	−.21	−.12	−.02	−.03	−.01

ATR: anterior thalamic radiation L; CS: corticospinal tract; CC: cingulum/cingulate gyrus; Cing_h: cingulum/hippocampus; IFO: inferior fronto-occipital fasciculus; ILF: inferior longitudinal fasciculus; SLF: superior longitudinal fasciculus; UN: uncinate fasciculus; SLF_t: superior longitudinal fasciculus temporal part; iM: individual mean; iSD: individual standard deviation; FA: fractional anisotropy. Tracks in italic are those showing a significant age effect.

*P < .05; **P < .01.
